# BRUCE liver-KO enhances MASLD/MASH development in the steatotic PTEN-KO background by impairing mitochondrial metabolism and activating STAT3

**DOI:** 10.1038/s41419-025-08294-5

**Published:** 2025-12-11

**Authors:** Lixiao Che, Camille K. Stevenson, David R. Plas, Jiang Wang, Chunying Du

**Affiliations:** 1 Departments of Cancer and Cell Biology, University of Cincinnati College of Medicine, Cincinnati, OH USA; 2 Pathology and Laboratory Sciences, University of Cincinnati College of Medicine, Cincinnati, OH USA

**Keywords:** Molecular biology, Cell biology

## Abstract

The IAP BRUCE (*Birc6*) plays multifaceted roles in apoptosis inhibition, DNA repair, and autophagy. Metabolic dysfunction-associated steatotic liver disease (MASLD) and its progressive form, metabolic dysfunction-associated steatohepatitis (MASH), affect up to 30% of the global population, yet their pathogenic mechanisms remain poorly understood. Given BRUCE’s high expression in healthy livers and its downregulation in MASLD/MASH, we investigated its functional role using liver-specific BRUCE-KO and BRUCE/PTEN dual liver-KO (DKO) mouse models. PTEN liver-KO provides a steatotic background and reflects a major etiological factor. By 3 months, BRUCE liver-deficiency alone induces MASLD onset, but when combined with PTEN liver-KO, increases DNA damage, apoptosis, oxidative stress, and MASLD-to-MASH progression, characterized by inflammation and fibrosis. Mechanistically, PTEN liver-KO is known to drive hepatosteatosis through AKT-mediated de novo lipogenesis (DNL) (PTEN-KO → AKT → DNL). However, BRUCE liver-KO impairs mitochondrial fatty acid -oxidation (FAO), respiration and ATP generation (BRUCE→Mitochondria→FAO). This distinct yet complementary mechanism of action leads to a ‘double hit’ of excessive lipid synthesis (PTEN-KO) and impaired lipid clearance (BRUCE-KO), exacerbating steatosis. To elucidate the molecular driver of MASH progression, we found both deficiencies converge on STAT3 activation, a central regulator of inflammation and fibrosis. Further, targeted inhibition of STAT3 with TTI-101 alleviates MASH, establishing a BRUCE/PTEN-STAT3 pathogenic axis. Notably, this axis is conserved in humans, with co-downregulation of BRUCE and PTEN and concurrent STAT3 activation in MASLD/MASH patient specimens. Collectively, these findings establish BRUCE as a key metabolic safeguard against MASLD/MASH, reveal cooperative hepatoprotection by BRUCE/PTEN against MASLD/MASH, and suggest STAT3 inhibition as a targeted therapeutic strategy for BRUCE/PTEN-deficient patients.

## Introduction

BRUCE (BIR repeat-containing ubiquitin-conjugating enzyme) initially identified as an inhibitor of apoptosis protein (IAP), has various roles in apoptosis regulation, DNA repair, and autophagy. BRUCE is an E3 ubiquitin ligase and scaffold protein. It functions to ubiquitinate a variety of pro-apoptotic proteins for their degradation via the ubiquitin-proteasome system to suppress apoptosis [1–6]. We have reported BRUCE whole-body mutant mice triggers p53-dependent apoptosis leading to embryonic lethality [7]. Beyond apoptosis regulation, BRUCE safeguards genomic stability by facilitating the repair of DNA double-strand breaks through the USP8-BRIT1-SWI/SNF pathway. Specifically, BRUCE promotes USP8-mediated deubiquitination of BRIT1, enabling chromatin remodeler SWI-SNF to decondense chromatin and subsequent facilitation of ATM/ATR recruitment to DNA damage sites, enhancing homologous recombination (HR) repair [8, 9]. This function is critical for male germ cell genome stability where BRUCE promotes the repair of physiological DNA breaks generated during spermatogenesis in mice [10]. We also found a key role of BRUCE in the regulation of cellular energy metabolism by modulating the AMPK-ULK1-mediated autophagy pathway. Under normal conditions, BRUCE suppresses autophagy; however, its depletion reduces ATP production and triggers autophagy as a compensatory mechanism to restore energy and nutrient supply [11]. Further studies by others have uncovered key molecular mechanisms and protein structural basis underlying BRUCE’s inhibitory effect on autophagy and apoptosis [12–15].

Metabolic dysfunction-associated steatotic liver disease (MASLD), distinguished by hepatic fat accumulation, can progress to its severe form, metabolic dysfunction-associated steatohepatitis (MASH), characterized by steatosis combined with inflammation with or without fibrosis. MASLD/MASH can further progress to hepatocellular carcinoma (HCC). Both MASLD and MASH are epidemic liver diseases and their pathogenic mechanisms remain largely unknown [16]. MASLD is present in up to 30% of the population and 175 million people have MASH to date, which is estimated to reach 200 million by 2030 [17]. Our studies have shown BRUCE is a suppressor of liver genomic instability, fibrosis, and HCC through DNA repair promotion, with clinical relevance demonstrated by its downregulation in 84% of HCC and 50% of MASLD/MASH cases [18, 19].

The current study investigates the functional role of BRUCE in MASLD/MASH using PTEN-KO mice as the hepatosteatosis background. Literature has documented PTEN dysfunction (downregulation, mutations, etc.) in liver disease patients. *Pten* mutations are an infrequent event in hepatocytes [20, 21], whereas reduction or absence in PTEN protein expression occurs in about 50% of primary hepatoma patients [22]. We selected a PTEN liver-KO steatotic mouse model instead of Western diet feeding models for key advantages: (1) PTEN loss either through genetic mutations or epigenetic silencing is a well-established etiological factor in human liver disease that reliably induces steatosis without dietary variability [23]; (2) PTEN inhibits AKT activity whereas loss of PTEN unleashes the inhibition and AKT overactivation leads to excessive lipogenesis and steatosis [24]. This well-defined PTEN/PI3K/AKT pathway allows clear mechanistic studies compared to the complex, poorly characterized effects of dietary components [25]; (3) PTEN knockout produces consistent, reproducible metabolic phenotypes unaffected by feeding variations among individual mouse [26]; (4) Western diets can reduce PTEN expression, so PTEN knockout reliably recapitulates Western diet effects [27]; 5) clinical MASLD/MASH biopsies show PTEN downregulation and concurrent hyperactivation of AKT, making this model more translationally relevant [27]. This study identifies BRUCE as a critical co-suppressor of MASLD/MASH, working synergistically with PTEN to regulate MASLD/MASH progression via a STAT3 central signaling hub that orchestrates the transition from steatosis to inflammatory fibrosis, while providing rigorous mechanistic insights.

## Materials and methods

### Animal studies

All animal studies followed IACUC-approved protocols. Liver-specific BRUCE-KO (BKO) mice were generated as previously described [18]. PTEN liver-KO (PKO) mice were created by crossing *Pten*^fl/fl^ (JAX #006440) with *Alb-Cre* (JAX #018961) and the liver DKO mice by crossing BKO with PKO mice. Liver ablation of protein expressions was confirmed by Western Blot using Cre-negative DKO littermates as WT controls. Experiments used 2-3-month-old male mice. For STAT3 inhibition, 2-month-old WT and DKO mice received daily intraperitoneal injection of TTI-101 (MedChemExpress HY-112288; 100 mg/kg) for three weeks. Sample size estimation was based on published literature and preliminary data. Mice were grouped randomly and the investigators were not blinded to the group allocations of the animals during the experiments. A minimum of three biological replicates (*n* = 3) per genotype and per treatment were used to ensure reproducibility and statistical rigor. No animals were excluded from the analysis.

### Liver histopathology

Mouse livers were fixed in 10% neutral buffered formalin, paraffin embedded and sectioned at 6 µm. Slides were stained with hematoxylin and eosin (H&E) for histology, Sirius Red solution (0.1% Sirius Red F3B in saturated picric acid) for collagen, and TUNEL (Millipore S7110) for cell death per manufacturer’s instructions.

For immunohistochemistry (IHC), deparaffinized liver slides underwent antigen retrieval using either sodium citrate buffer (pH 6.5) or EDTA (pH 8.0), followed by blocking of endogenous peroxidase (3% H_2_O_2_), and serum (5% normal goat serum/PBS containing 0.1% Triton X-100). Sections were incubated with primary antibodies overnight at 4 °C, followed by detection using Vectastain ABC kit (Vector Laboratories PK-6100) and DAB substrate (Sigma D3939). Secondary antibodies application and hematoxylin counterstaining were performed according to the manufacturers’ protocols.

Mouse livers were OCT-embedded, frozen on dry ice, and sectioned at 10 µm. Sections were stained with Oil Red O (0.3% in 60% isopropanol, 5 min at room temperature (RT) for neutral lipids, or Dihydroethidium (DHE, 2 µM, 37 °C for 30 min; Invitrogen D11347) for superoxide.

All slides were analyzed microscopically and quantified using ImageJ.

### Triglyceride (TG) content

Liver triglycerides were quantified by a luciferase-based assay as described by the manufacturer’s instructions (Promega J3160).

### Primary hepatocyte isolation

Primary hepatocytes were isolated from anesthetized mice by IVC perfusion (25-gauge cannula; Fisher 02-664-2) with a portal vein incision, following established protocols [28], with viability (>90%) confirmed by trypan blue exclusion.

### Seahorse mitochondrial function analysis

Primary hepatocytes from 3-month-old male mouse livers were plated (3 × 10⁴ cells/well) in Williams E Medium (Thermo 12551032) containing 10% FBS, antibiotics (2%), and metabolic supplements (1% each of pyruvate, glutamine, Insulin/transferrin/selenium) in Seahorse XF96 plates (Agilent 103794-100). After 24 h, cells received BSA or BSA-palmitate (100 µM; Cayman 10006627) for 48 h. Seahorse XFe96 measured OCR in assay medium (10 mM glucose, 1 mM pyruvate, 2 mM glutamine) following sequential injection of mitochondrial modulators (4 µM etomoxir, 1.5 µM oligomycin, 1 µM FCCP, 0.5 µM rotenone/antimycin A) with Hoechst 33342 (2.5 µM; Fisher 51-17) for cell number normalization by CLARIOstar (BMG Labtech). Fatty acid oxidation was assessed using FAO stress test kit according to manufacturer’s instruction (Agilent 103672-100). Wave (Agilent Technologies) was used for data acquisition and quantification.

### Determination of mitochondrial numbers in primary hepatocytes

Primary hepatocytes isolated from 3-month-old male mouse livers were seeded in 24-well plates with cover slips inside. After 24 h, cells were fixed in 4% PFA, followed by permeabilization with 0.1% Triton X-100. Cells were then blocked with 5% normal goat serum in PBS, incubated with an antibody specific to TOM20 overnight at 4 °C, and an Alexa Fluor™ 594-conjugated secondary antibody for 1 h at RT. Cells were imaged by confocal microscopy and mitochondrial numbers per cell were quantified as total TOM20 fluorescence intensity using Fiji. TOM20 is an outer mitochondrial membrane protein with a direct correlation between its immunofluorescence and the number of mitochondria present in a cell or tissue.

### Western blot (WB)

Liver tissues were homogenized in RIPA buffer (20 mM Tris-HCl pH 8.0, 150 mM NaCl, 2 mM EDTA, 1% NP-40, 1% sodium deoxycholate, 0.1% SDS) supplemented with protease inhibitors (Thermo 88668) and universal nuclease (Thermo 88700). After centrifugation (12,000 × *g*, 4 °C, 10 min), supernatants were collected as liver protein lysates. For WB, 50 μg protein lysates were denatured, separated by SDS-PAGE, and transferred to nitrocellulose membranes. Membranes were blocked with 3% non-fat dry milk in PBST, then incubated with primary antibodies (4 °C, overnight) and secondary antibodies (RT, 1 h). Proteins were detected by ECL (Revvity NEL103001EA) and visualized with either a ChemiDoc system (Bio-Rad) or X-ray film. Original WBs are provided in the Supplemental Material.

### RNA analysis

Total RNA was extracted from liver tissue using the mirVana™ miRNA Isolation Kit (Thermo AM1560). RNA-seq was processed by University of Cincinnati Genomics Core with Heatmaps generated with HemI 1.0 software. qPCR was performed using iScript cDNA Synthesis Kit (Bio-Rad 1708891) and SYBR Green Supermix (Bio-Rad 1725121) on a CFX Connect system (Bio-Rad 1855201), with GAPDH normalization.

Publicly available data from GEO (GSE126848) were analyzed via GEO2R to compare *Birc6* expressions along normal, MASLD, and MASH samples. A separate cohort (GSE162694; *n* = 143) provided expression data for *Birc6*, *Pten* and *Stat3* with corresponding fibrosis and NAS scores.

### Data analysis

Data are expressed as the means ± SEM. Statistical significance was determined by the two-tailed Student’s *t* test or 2-way ANOVA using GraphPad Prism with *p* < 0.05. n.s.: not significant, *p < 0.05, **p < 0.01, ***p < 0.001. Data meet the assumptions of the tests of normality and variance. Experiments were replicated at minimum two to three independent times. Sample size and replicate information are provided in the figure legends.

### Antibodies

BD Biosciences: BRUCE (611193 for IHC). Cell Signaling: Actin (3700), AKT (9272), phospho-AKT (Ser473) (pAKT-S473, 4060), β-Catenin (9582), cleaved Caspase-3 (9961), F4/80 (70076), GSK3β (9315), phospho-GSK3β (Ser9) (pGSK3β-S9, 9336), γH2AX (9718), Ki67 (12202), PTEN (9559), α-SMA (19245), SOX9 (82630), STAT3 (9139), phospho-STAT3 (Tyr705) (pSTAT3-Y705, 9145). DSHB: CK19 (Clone TROMA-III). Santa Cruz Biotechnologies: NIMP-R14 (sc-59338), TOM20 (sc-11415). Sigma: BRUCE (PLA0098 for Western Blot), Tubulin (T9026). Thermo: Alexa Fluor™ 594 (A-11012), Vinculin (Clone 42H89L44).

### qRT-PCR primers

*GAPDH*: forward CAAAATGGTGAAGGTCGGTGTG and reverse TGATGTTAGTGGGGTCTGGCTC.

*FASN*: forward GCTGCGGAAACTTCAGGAAAT and reverse AGAGACGTGTCACTCCTGGACTT. *ACLY*: forward GGTGTCAACGAACTGGCGAA and reverse GTTTGCAATGCTGCCTCCAA.

## Results

### BRUCE liver-KO exacerbates MASLD in steatotic background provided by PTEN liver-KO

**Fig. 1:**
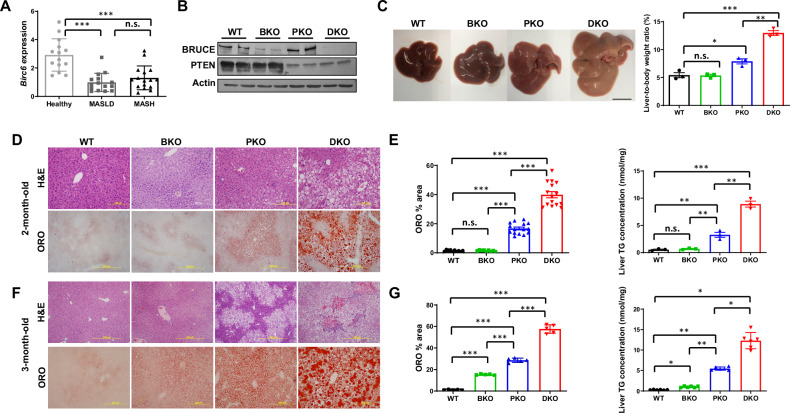
BRUCE liver-KO accelerates liver MASLD in the steatotic PTEN liver-KO background. *Birc6* expressions in healthy, MASLD and MASH livers were analyzed by GEO2R in the GSE126848 dataset (**A**). Western Blot (WB) analysis of indicated proteins with liver homogenates from 2-month-old mice (**B**), their liver images (**C**, left), and liver-to-body weight ratio (**C**, right); scale bar 1 cm. H&E analysis (upper) and ORO staining (lower) of liver tissue sections (**D**) with both ORO and total liver TG content quantified (**E**) in 2-month-old mice, which were repeated with 3-month-old mice (**F**, **G**). *p < 0.05, **p < 0.01, ***p < 0.001 by student’s *t* test. Healthy human livers (*n* = 14), MASLD (*n* = 15), MASH (*n* = 16) including both male and female subjects. Mice per genotype (*n* = 3) with representative images quantified (*n* = 3-15).

We have reported BRUCE downregulation in 50% MASLD/MASH cases by BRUCE IHC analysis of patient livers [18, 19]. To increase the rigor of this correlation, we analyzed transcriptome signatures of human MASLD and MASH liver samples (GSE126848), which confirmed significant *Birc6* downregulation (Fig. 1A). We therefore established BRUCE liver-KO (BKO) alone or with PTEN liver-KO background (DKO) with protein ablation confirmed by Western Blot (WB) and notably, loss of either BRUCE or PTEN did not affect the expression of the other (Fig. 1B, see original Supp. Material). At 2-3 months, while PTEN liver-KO (PKO) livers exhibited expected steatotic features (increased size, pallor, and higher liver-to-body weight ratio) [26, 29], BKO mice showed normal liver morphology and liver-to-body weight ratio (Fig. 1C). Strikingly, DKO livers displayed the most severe phenotypes of largest size, palest coloration, and highest liver-to-body weight ratio (Fig. 1C). Histological examination of PKO mouse livers revealed expected steatosis, evident by Oil Red O staining of hepatic neutral lipids as early as 2 months of age (Fig. 1D, E) with progression to more severe steatosis by 3 months (Fig. 1F, G). In contrast, BKO mouse livers showed minor structural disorganization but no steatosis at 2 months (Fig. 1D, E) and mild steatosis by 3 months (Fig. 1F, G), marking MASLD onset in BKO mice (Fig. 1D–G). Remarkably, DKO displayed the most severe phenotypes at both ages, with mixed steatosis/ballooning (Fig. 1D–G), demonstrating BRUCE deficiency accelerates MASLD in PTEN-deficient livers, with the 2–3-month timepoint capturing disease onset in BKO and exacerbation in DKO.

### BRUCE-KO drives hepatic steatosis through impaired mitochondrial functions

**Fig. 2:**
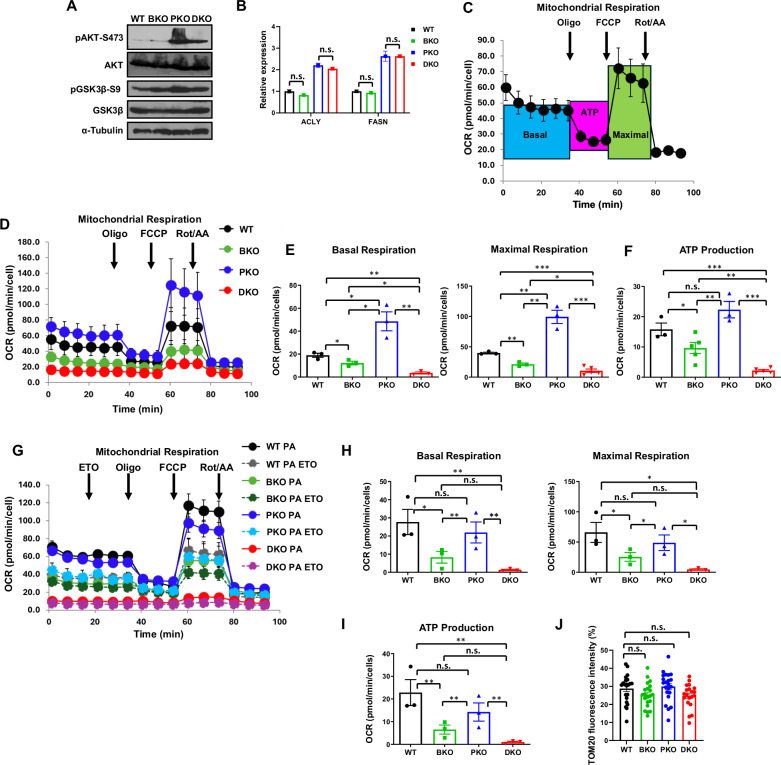
BRUCE-KO hepatocytes show mitochondrial metabolic dysfunction. WB of whole liver homogenates for indicated proteins from 2-month-old mice (**A**). qRT-PCR of lipogenic genes (*ACLY* and *FASN)* (**B**). Diagram of basal, maximal respiration, and ATP production measured by Seahorse assay, indicating time points of mitochondrial inhibitor injections (e.g., oligomycin, FCCP, rotenone/antimycin A) (**C**). Mitochondrial respiration kinetics of primary hepatocytes from 3-month-old mice (**D**) and their basal (**E**; left), maximal (**E**; right) respiration, and ATP production (**F**). Seahorse FAO assay showing basal and maximal respiration and ATP production after 24 h palmitic acid (PA) treatment (100 µM, BSA-conjugated) with FAO inhibitor (Etomoxir/ETO) and mitochondrial inhibitor injections (**G**). Oxygen consumption rate (OCR) differences (PA minus PA + ETO) were used to calculate basal (**H**, left), maximal (**H**, right) respiration, and ATP production from exogenous FAO (**I**). Quantification of TOM20 immunofluorescence intensity, proportional to mitochondrial number, from confocal images of each primary hepatocyte genotype immunostained with an anti-TOM20 antibody (**J**). n.s.: not significant, *p < 0.05, **p < 0.01, ***p < 0.001 by student’s *t* test. Mice per genotype for qRT-PCR (*n* = 3). Seahorse technical replicates (*n* = 3–5). Primary hepatocytes imaged for TOM20 immunofluorescence intensity/mitochondrial number analysis (*n* = 20–22). Oligo=oligomycin, FCCP=Carbonyl cyanide p-trifluoromethoxyphenylhydrazone, Rot/AA=rotenone/antimycin a.

PTEN deficiency drives steatosis via PI3K-AKT-mediated DNL [23, 30, 31]. In PTEN-KO mice, we confirmed PI3K-AKT activation (increased pAKT-S473 and its substrate pGSK3β-S9) and elevated key DNL gene expression (*ACLY* and *FASN)* (Fig. 2A, B, see original WB in Supp. Material). However, BRUCE-KO mice showed no such changes (Fig. 2A, B), indicating BRUCE-KO does not affect these lipogenic gene expressions at this time point.

Beyond lipogenesis, mitochondrial dysfunction is a known contributor to MASLD pathogenesis in patients [32–34]. Seahorse analysis found that while PTEN-KO hepatocytes showed elevated respiration, consistent with literature [35], BRUCE-KO hepatocytes had significantly impaired basal/maximal respiration and ATP production versus WT (Fig. 2C–F). DKO cells exhibited the greatest suppression of mitochondrial function (Fig. 2C–F). Palmitic acid (PA) oxidation assays confirmed markedly reduced FAO in BRUCE-KO and DKO hepatocytes (Fig. 2G–I), establishing BRUCE’s essential role in mitochondrial FAO. Notably, mitochondrial numbers remained unchanged across the four primary hepatocyte genotypes, as determined by TOM20 immunofluorescence quantification (Fig. 2J). Since TOM20 is an outer mitochondrial membrane protein whose fluorescence correlates with mitochondrial numbers and abundance [36], these findings indicate that the observed differences in respiration, ATP production, and FAO capacity reflect mitochondrial dysfunction rather than reduced mitochondrial number. Importantly, these findings reveal distinct and complementary mechanisms: PTEN suppresses AKT-lipogenesis (PTEN ┫AKT → DNL), while BRUCE promotes mitochondrial lipid clearance (BRUCE→Mitochondria→FAO). The observation that BKO and DKO livers differ in steatosis despite shared BRUCE loss can be explained by a ‘double-hit’ metabolic model. In BRUCE-KO mice, preserved PTEN activity limits lipogenesis resulting in mild MASLD from impaired FAO alone, or the ‘first hit.’ However, in DKO mice, failed lipid oxidation (BRUCE loss) combined with the ‘second hit’ of unrestrained DNL (PTEN loss) creates a metabolic vicious cycle, explaining both the initial steatosis in BKO and its dramatic exacerbation in DKO. This synergy underscores how coordinated regulation of lipid synthesis and disposal maintains hepatic metabolic homeostasis.

### BRUCE liver-KO exacerbates hepatic oxidative stress, DNA damage and apoptosis in steatotic background provided by PTEN liver-KO

**Fig. 3:**
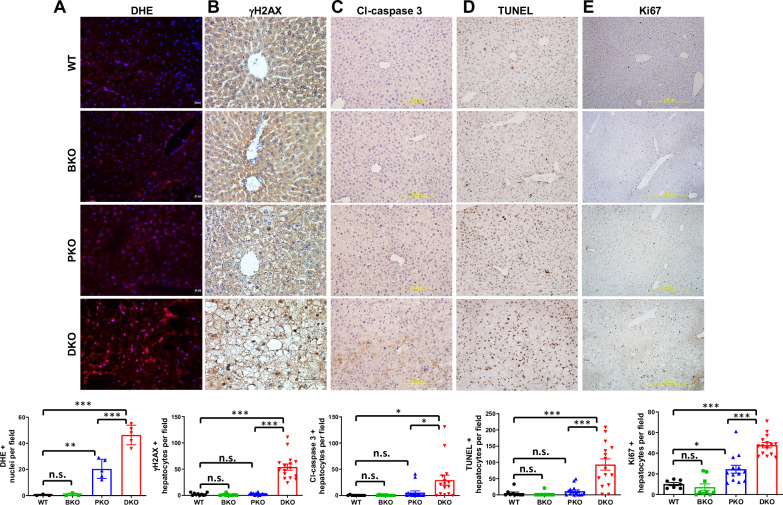
Exacerbated oxidative stress, DNA damage, cell death and regeneration in DKO mice. Liver sections from 2/3-month-old mice stained with dihydroethidium (DHE) for superoxide (**A**), IHC of γH2AX for DNA damage (**B**), cleaved caspase-3 (Cl-caspase-3) for apoptosis (**C**), TUNEL for cell death (**D**), and Ki67 for proliferation (**E**), with quantification shown below each panel. n.s.: not significant, *p < 0.05, **p < 0.01, ***p < 0.001 by student’s *t* test. Mice per genotype (*n* = 3) with representative images quantified (*n* = 5–15).

Given the tight link between mitochondrial dysfunction and oxidative stress in MASLD/MASH patients [37, 38], we measured superoxide levels using DHE fluorescence. It revealed progressive accumulation of superoxide of nearly minimal in BKO, moderate in PKO, and severe in DKO livers (Fig. 3A), which mirrors the increasing steatotic toxicity across these models. While BRUCE normally mediates DNA repair and inhibit apoptosis [7–9], only DKO (but not BKO) livers exhibited significant DNA damage (γH2AX), apoptosis (cleaved caspase-3/TUNEL; Fig. 3B-D), and reparative/compensatory proliferation (Ki67; Fig. 3E) [39]. This aligns with the ‘double-hit’ metabolic model: In BKO mice, preserved PTEN function maintains lipid and redox homeostasis below the threshold for irreversible damage, despite BRUCE deficiency. In stark contrast, DKO mice suffer synergistic metabolic collapse from the two metabolic hits from BRUCE and PTEN loss, creating steatotic toxicity. The resulting oxidative overload, combined with loss of BRUCE’s protective functions, permits unchecked DNA damage, hepatocyte apoptosis, and reparative/compensatory proliferation. These findings demonstrate how BRUCE and PTEN cooperatively maintain hepatic homeostasis through complementary pathways: PTEN regulates lipid input, while BRUCE controls lipid disposal and damage mitigation.

### BRUCE liver-KO accelerates MASLD-to-MASH progression in DKO mice

**Fig. 4:**
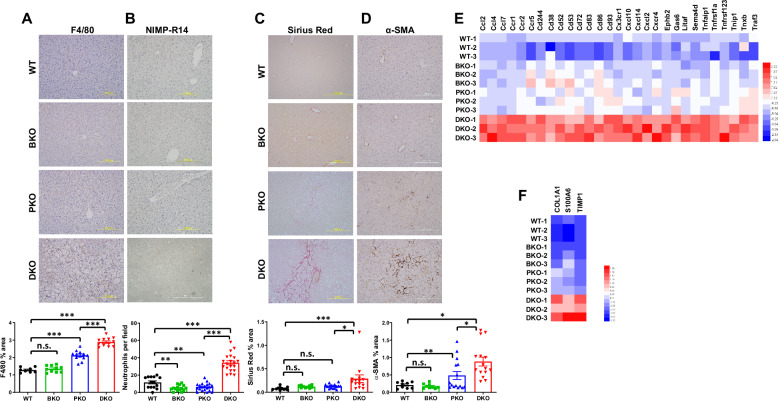
BRUCE-KO accelerates inflammation and fibrosis in PTEN-KO background. IHC of liver sections from 2/3-month-old mice for F4/80 (**A**), NIMP-R14 (**B**), Sirius Red (**C**), and α-SMA (**D**) with quantification below. RNA-seq heatmaps showing pro-inflammatory (**E**) and HSC activation genes (**F**) across four mouse models. n.s.: not significant, *p < 0.05, **p < 0.01, ***p < 0.001 by student’s *t* test. Mice per genotype (*n* = 3) wi*t*h representative images quantified (*n* = 3–16). Mice per genotype for RNA-seq (*n* = 3).

**Fig. 5:**
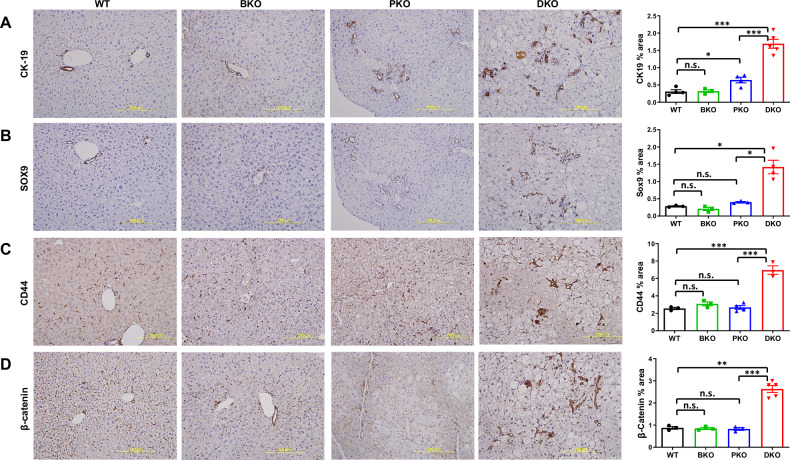
BRUCE-KO promotes crucial events involved in hepatic inflammation and fibrosis. IHC of liver sections from 2/3-month-old mice for CK-19 (**A**), SOX9 (**B**), CD44 (**C**) and β-catenin (**D**) with quantification to the righthand side. *p < 0.05, ***p < 0.001 by student’s *t* test. Mice per genotype (*n* = 3) with representative images quantified (*n* = 8–20).

Given the elevated oxidative stress in DKO livers, we conducted a comprehensive analysis of downstream MASH pathological consequences using established MASLD-to-MASH progression markers. We found DKO livers displayed pronounced inflammation indicated by immune cell infiltration (macrophages F4/80+ and neutrophils NIMP-R14 + ; Fig. 4A, B) and robust fibrogenesis evidenced by α-SMA+ hepatic stellate cell (HSC) activation and Sirius Red+ collagen deposition (Fig. 4C, D). Pro-inflammatory and fibrogenic pathway activation was transcriptomically confirmed at gene expression levels (Fig. 4E, F). The MASLD-to-MASH progression was further validated by CK19-postive biliary expansion indicating chronic injury, upregulation of fibrogenic regulators SOX9 for ECM production [40], CD44 for macrophage polarization/HSC activation [41, 42], and β-catenin for fibrosis progression signaling [43] (Fig. 5A–D). Strikingly, while PTEN-KO mice typically develop MASH by 10 months [26, 29], BRUCE deficiency accelerated this to just 2–3 months in DKO mice, a 4x acceleration, again demonstrating their synergistic roles in disease pathogenesis through distinct but complementary mechanisms.

### The BRUCE/PTEN-STAT3 axis drives MASLD-to-MASH progression in DKO mice

**Fig. 6:**
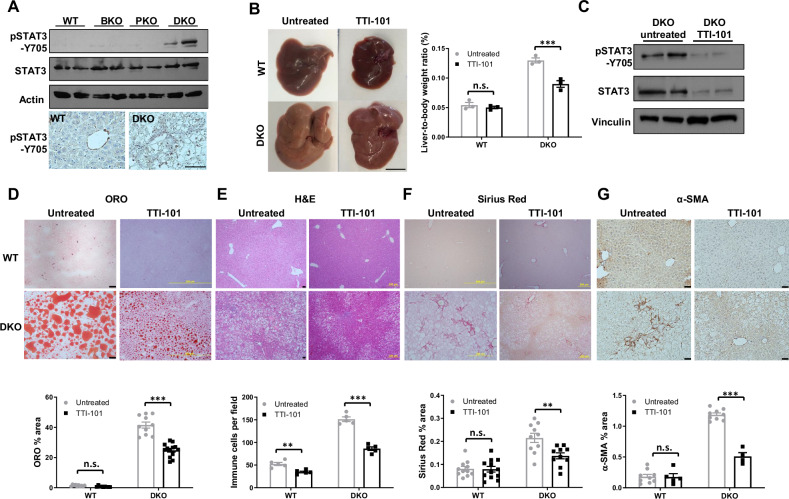
STAT3 activation and in vivo inhibition of STAT3 ameliorates MASH in DKO mice. **A** WB of liver lysates from 2-month-old mice (upper) and IHC of pSTAT3-Y705 with WT and DKO liver sections showing nuclear positivity of pSTAT3-Y705 both in hepatocytes and NPCs in DKO (lower); scale bar 50 µm. **B** Liver images of WT and DKO mice treated with TTI-101 (100 mg/kg/bw, daily i.p.) for 3 weeks or left untreated with liver-to-body weight ratio quantified; scale bar 1 cm. These mice were analyzed by WB for indicated proteins (**C**), ORO (**D**), H&E (**E**), Sirius Red (**F**) and IHC for α-SMA, scale bar 50 µm (**G**) with quantification below. n.s.: not significant, **p < 0.01, ***p < 0.001 by 2-way ANOVA. Mice per genotype (*n* = 3) with representative images quantified (*n* = 4–11).

**Fig. 7:**
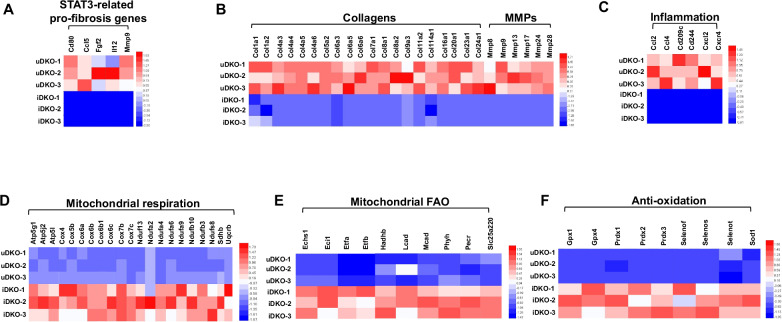
The anti-MASLD/MASH effects of STAT3-specfic inhibitor TTI-101 at gene expression levels. RNA-seq heatmaps showing STAT3-related pro-fibrosis (**A**), pro-fibrotic collagen and MMP (**B**), pro-inflammatory (**C**), mitochondrial respiration (**D**), FAO (**E**), and antioxidant genes (**F**). (uDKO untreated DKO, iDKO inhibitor treated DKO). Mice per treatment (*n* = 3).

STAT3, a transcription factor regulating inflammation and fibrosis [44, 45], requires phosphorylation at Y705 (pSTAT3-Y705) for activation [46, 47]. Since AKT activation and mitochondrial dysfunction can trigger STAT3 activation [47, 48], we compared its activation among the four genotypes. WB revealed elevated pSTAT3-Y705 exclusively in DKO mice, while total STAT3 remained unchanged (Fig. 6A, upper, see original WB in Supp. Material). IHC confirmed pSTAT3-Y705 activation in the nuclei of hepatocytes and non-parenchymal cells (NPCs) (Fig. 6A, lower). To establish causality, we used TTI-101, a selective STAT3 inhibitor targeting its SH2 domain [49, 50], without mitochondrial toxicity in clinical trials [51, 52]. TTI-101 treatment in DKO mice (100 mg/kg, 3 weeks) reduced hepatomegaly (Fig. 6B), decreased active/total STAT3 (Fig. 6C, see original WB in Supp. Material), and improved steatosis (Fig. 6D), inflammation (immune cell infiltration; Fig. 6E), and fibrosis (Fig. 6F, G). Transcriptomics confirmed TTI-101 downregulation of STAT3-regulated pro-fibrotic genes [53], extracellular matrix regulators (collagens/matrix metalloproteinases [MMPs]), and inflammatory mediators (Fig. 7A–C), while restoring mitochondrial respiration, FAO, and antioxidant genes (Fig. 7D–F). These results define a new ‘BRUCE/PTEN-STAT3’ axis, where STAT3 hyperactivation drives MASH progression in BRUCE/PTEN-deficient livers. The discoveries of BRUCE/PTEN dual deficiencies converging on STAT3 hyperactivation and STAT3 inhibition ameliorating MASH reveal mechanistic and therapeutic advance in MASLD/MASH.

### Clinical relevance of the BRUCE/PTEN-STAT3 axis

**Fig. 8:**
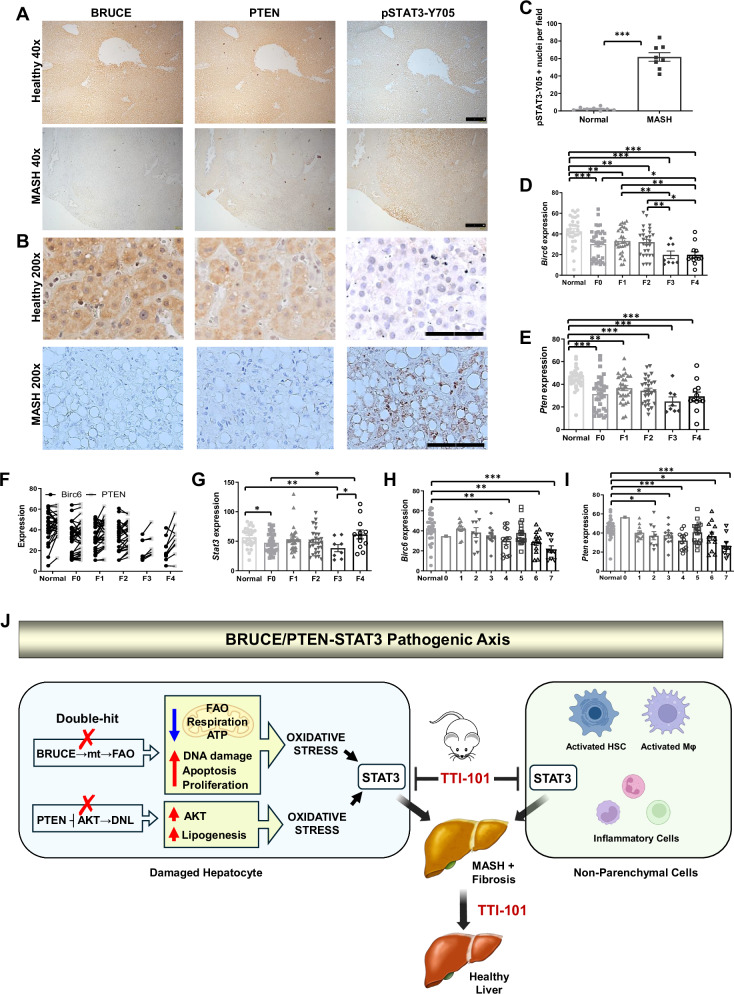
Clinical relevance of dual downregulation of BRUCE and PTEN in MASH patient specimens and a working model. IHC of BRUCE, PTEN and pSTAT3-Y705 in consecutive liver sections from healthy individuals and MASH specimens at 40x (**A**, scale bar 400 µm) and 200x magnification (**B**, scale bar 100 µm) shows nuclear pSTAT3-Y705 positivity only in MASH livers, with quantification shown (**C**). *Birc6* (**D**) and *Pten* (**E**) expression across normal livers and MASH fibrosis stages F0-F4 were analyzed using GEO2R, with *Birc6* and *Pten* co-expression (**F**) *and Stat3* expression (**G**) shown. *Birc6* (**H**) and *Pten* (**I**) expression in normal and MASH livers with varying NAS scores were also analyzed. All data were from the GSE162694 dataset. (**J**) A model illustrating BRUCE/PTEN-STAT3 axis in MASLD/MASH regulation: The combined loss of BRUCE (BRUCE→mt→FAO) and PTEN (PTEN┫AKT → DNL) creates a ‘double hit’ that exacerbates MASLD. BRUCE deficiency impairs mitochondrial metabolism (specifically, fatty acid oxidation, respiration, and bioenergetics), while PTEN loss activates the AKT pathway, promoting de novo lipogenesis. Together, these defects intensify hepatic lipid accumulation, driving MASLD. Additionally, BRUCE loss increases DNA damage, apoptosis, and compensatory hepatocyte proliferation, further elevating oxidative stress. This cumulative oxidative stress, amplified by AKT-driven oxidative stress signals, triggers STAT3 activation in both hepatocytes and non-parenchymal cells, driving progression to MASH with fibrosis, which can be mitigated by STAT3 inhibitor TTI-101. Yellow-highlighted areas represent steatosis-promoting events. mt: mitochondria. ***p < 0.001 by student’s *t* test. Normal (*n* = 5), MASH (*n* = 4), with representative images quantified (*n* = 8). Normal histology (*n* = 33), F0 (*n* = 33), F1 (*n* = 30), F2 (*n* = 27), F3 (*n* = 8), F4 (*n* = 12).

To assess clinical relevance, we analyzed BRUCE/PTEN and pSTAT3-Y705 expression in human liver specimens. IHC revealed an inverse correlation: healthy controls showed strong cytoplasmic BRUCE/PTEN with minimal nuclear pSTAT3-Y705, whereas MASH samples exhibited BRUCE/PTEN downregulation alongside nuclear pSTAT3-Y705 accumulation in hepatocytes and NPCs (40x; Fig. 8A). High-resolution imaging (200x) confirmed this reciprocity: BRUCE/PTEN-rich areas lacked pSTAT3-Y705, whereas BRUCE/PTEN-deficient regions showed prominent nuclear pSTAT3-Y705 (Fig. 8B). Quantification confirmed elevated nuclear pSTAT3-Y705 in MASH, particularly in BRUCE/PTEN-low zones (Fig. 8C).

Transcriptomic analysis of human MASLD livers (GSE162694) revealed co-progressive downregulation of *Birc6* and *Pten* across fibrosis stages (F0-F4) versus normal histology (Fig. 8D, E), and their dual downregulation was largely found in the same patient (Fig. 8F). Notably, STAT3 mRNA levels remained mostly unchanged, consistent with post-translational activation (phosphorylation) rather than transcriptional regulation (Fig. 8G). Reduced *Birc6/Pten* correlated with increasing severity of the pathology reflected by NAFLD Activity Scores (NAS) (Fig. 8H, I), clinically validating our murine model and confirming cross-species conservation of the BRUCE/PTEN-STAT3 axis in MASH progression.

## Discussion

Our findings establish a conserved “BRUCE/PTEN-STAT3” pathogenic axis in human and murine livers governing MASLD/MASH progression, in which BRUCE and PTEN play a cooperative hepatoprotection function through distinct yet complementary pathways: BRUCE maintains mitochondrial FAO and bioenergy homeostasis (BRUCE→Mitochondria (mt)→FAO), while PTEN suppresses AKT-driven lipogenesis (PTEN ┫AKT → DNL). Under physiological conditions, the two pathways synergistically limit hepatic steatosis. However, BRUCE and PTEN dual deficiency acts as ‘double-hit’ creating an amplified pathological cascade - BRUCE loss drives steatosis via impaired mitochondrial FAO, while PTEN loss drives lipid accumulation via unrestrained AKT activation. Further, BRUCE loss elevates oxidative stress via increasing DNA damage and apoptosis, while PTEN loss elevates oxidative stress via AKT-mediated upregulation of genes that produce ROS. Together, they generate overwhelming oxidative stress, which hyperactivates STAT3 in both hepatocytes and NPCs. Suppression of MASLD/MASH by STAT3 inhibitor TTI-101 establishes STAT3 hyperactivation as a central molecular switch converting metabolic stress into inflammation and fibrosis, explaining the rapid MASLD-to-MASH transition observed in BRUCE and PTEN dual deficient livers (Fig. 8J).

### Significance of BRUCE being a new repressor of MASLD/MASH

The discovery of BRUCE as a multifunctional MASH suppressor represents a paradigm shift in understanding disease progression, as it uniquely integrates three fundamental protective mechanisms: (1) mitochondrial metabolism regulation (maintaining FAO and ATP production to prevent steatosis and oxidative stress); (2) genomic stability maintenance (repairing oxidative DNA damage); and (3) cell viability (blocking apoptosis and cell death-induced release of pro-inflammatory Damage-Associated Molecular Patterns (DAMPs)). This trifunctionality explains why BRUCE deficiency creates a perfect storm of metabolic dysfunction, cellular damage, and inflammatory signaling - uniquely positioning it to orchestrate the transition from simple steatosis to progressive MASH. Unlike single-pathway regulators, BRUCE-deficiency induced simultaneous collapse of lipid clearance, DNA repair, and cell survival mechanisms creates an irreversible tipping point, characterized by self-sustaining fibro-inflammation under steatotic background provided by PTEN KO. This explains the dramatic acceleration of MASH in DKO mice and highlights BRUCE as a master regulatory node whose pleiotropic functions make it indispensable for hepatic homeostasis. BRUCE emerges as a central guardian against MASH by integrating metabolic, genomic, and survival pathways. Its pleiotropic functions make it a unique therapeutic node. This work redefines MASH as a disease of coupled metabolic and cellular stress responses, opening new avenues for mechanism-based therapies.

### Impact of the BRUCE/PTEN-STAT3 pathogenic axis on MASLD/MASH pathogenesis and therapy

The discoveries that BRUCE/PTEN dual deficiency converges on STAT3 hyperactivation and that inhibition of STAT3 ameliorates MASLD/MASH pathogenesis represent mechanistic, clinical, and therapeutic breakthroughs in MASLD/MASH research. *Mechanistically*, the uncovered central driver STAT3 emerges as a critical inflammatory/fibrotic switch integrating the stresses from dual BRUCE and PTEN loss into inflammation/fibrosis in MASLD-to-MASH progression, forcing synergistic overactivation of STAT3, and triggering a feedforward loop of hepatocyte injury, immune cell recruitment, and HSC activation. The accelerated MASH development in DKO mice (vs. single KOs) is now traceable to STAT3’s role as an amplifier of cellular stress signals. *Clinically*, the findings provide new insights into unsolved clinical questions of why only a subset of MASLD patients progress to MASH and the factors driving this selective progression remain unclear. The current study identifies BRUCE downregulation as a potential key determinant of that progression. Further, the biomarker triad of BRUCE ↓ /PTEN ↓ /pSTAT3-Y705↑ signature in human MASH specimens offers a molecular stratification tool for clinical trials and identifies a high-risk subgroup likely to benefit from STAT3-targeted therapy. *Therapeutically*, the study provides proof of concept for STAT3 inhibition (TTI-101) as being effective to ameliorate steatosis, inflammation, and fibrosis in DKO mice, confirming STAT3 as a druggable master regulator downstream of BRUCE/PTEN in murine MASH models. This provides precision medicine potential for patients with BRUCE/PTEN-low MASLD/MASH as they may be ideal candidates for STAT3 inhibitors therapy.

### Broader implications of this study

Our findings highlight a central role for STAT3 in driving the development of MASLD and MASH phenotypes in the context of BRUCE and PTEN deficiency. Pharmacological inhibition of STAT3 in vivo markedly ameliorated most of the liver pathology, underscoring STAT3 activation as a dominant mediator of steatohepatitis progression in this model. Nevertheless, we acknowledge that other signaling nodes likely contribute to the observed phenotypes. STAT1 has been implicated in MASH progression through promoting hepatic immune cell recruitment and inflammatory responses, whereas the JAK kinases, upstream regulators of both STAT1 and STAT3, may represent convergent drivers of inflammation and fibrogenesis [54]. Although we did not examine the phosphorylation status or functional contribution of STAT1 or JAK signaling in this study, it remains possible that their activation synergizes with STAT3 to exacerbate disease progression in BRUCE/PTEN-deficient livers.

### Future directions

This study opens multiple research avenues to further understand BRUCE’s role in MASLD/MASH pathogenesis. Mitochondrial dysfunction in BRUCE-deficient livers highlights the need for investigating how its enzymatic and scaffolding functions regulate mitochondrial metabolism via protein ubiquitination. In parallel, examining how mitochondrial-derived immune signals/mediators activate macrophages and hepatic stellate cells will reveal key mechanisms of disease progression. It is also critical to determine the specific DNA repair and apoptosis inhibition pathways involved in MASLD/MASH development governed by BRUCE. Our published findings that BRUCE depletion enhances autophagy through AMPK activation points to the need for determining the role of BRUCE-regulated hepatic autophagy in MASLD/MASH development. These mechanistic insights open multiple promising therapeutic directions. Strategic dissection of the phosphorylation status of STAT1 and JAK family members within the BRUCE/PTEN-STAT3 axis will determine whether selective inhibition of these pathways offers additive or distinct benefits beyond STAT3 blockade alone. Such work will refine our understanding of the STAT-JAK axis in MASLD/MASH and guide pathway-targeted intervention.

At present, only two drugs are FDA-approved for MASH. Rezdiffra (resmetirom), a thyroid hormone receptor-β (THR-β) agonist was approved in March 2024. Although it shows promise in clinical trials, it is efficacious for only 26–30% patients compared to 10% in the placebo group in the trials [55]. Wegovy (semaglutide), a GLP-1 receptor agonist approved in August 2025 for adults with moderate-to-advanced fibrosis, provides benefits partly through weight loss, but its mechanism in MASH remains incompletely understood [56]. Our results suggest that a combination therapeutic strategy of BRUCE activation, STAT3 inhibition, and selective modulation of the STAT-JAK axis, could complement existing metabolic approaches such as Rezdiffra and Wegovy. By jointly targeting mitochondrial metabolism, autophagy, and inflammatory signaling, this integrated approach may provide a more durable and comprehensive treatment paradigm for MASLD/MASH.

## Supplementary information

Supplemental Material
