# Supplementary material for: BRUCE liver-KO enhances MASLD/MASH development in the steatotic PTEN-KO background by impairing mitochondrial metabolism and activating STAT3
Source: Cell Death Dis. 2025 Dec 11;17(1):88. doi: 10.1038/s41419-025-08294-5 (PMC12830714; doi:10.1038/s41419-025-08294-5)
Supplement: Supplementary file 1 — Supplemental Material [file 41419_2025_8294_MOESM1_ESM.pptx]

## Slide 1
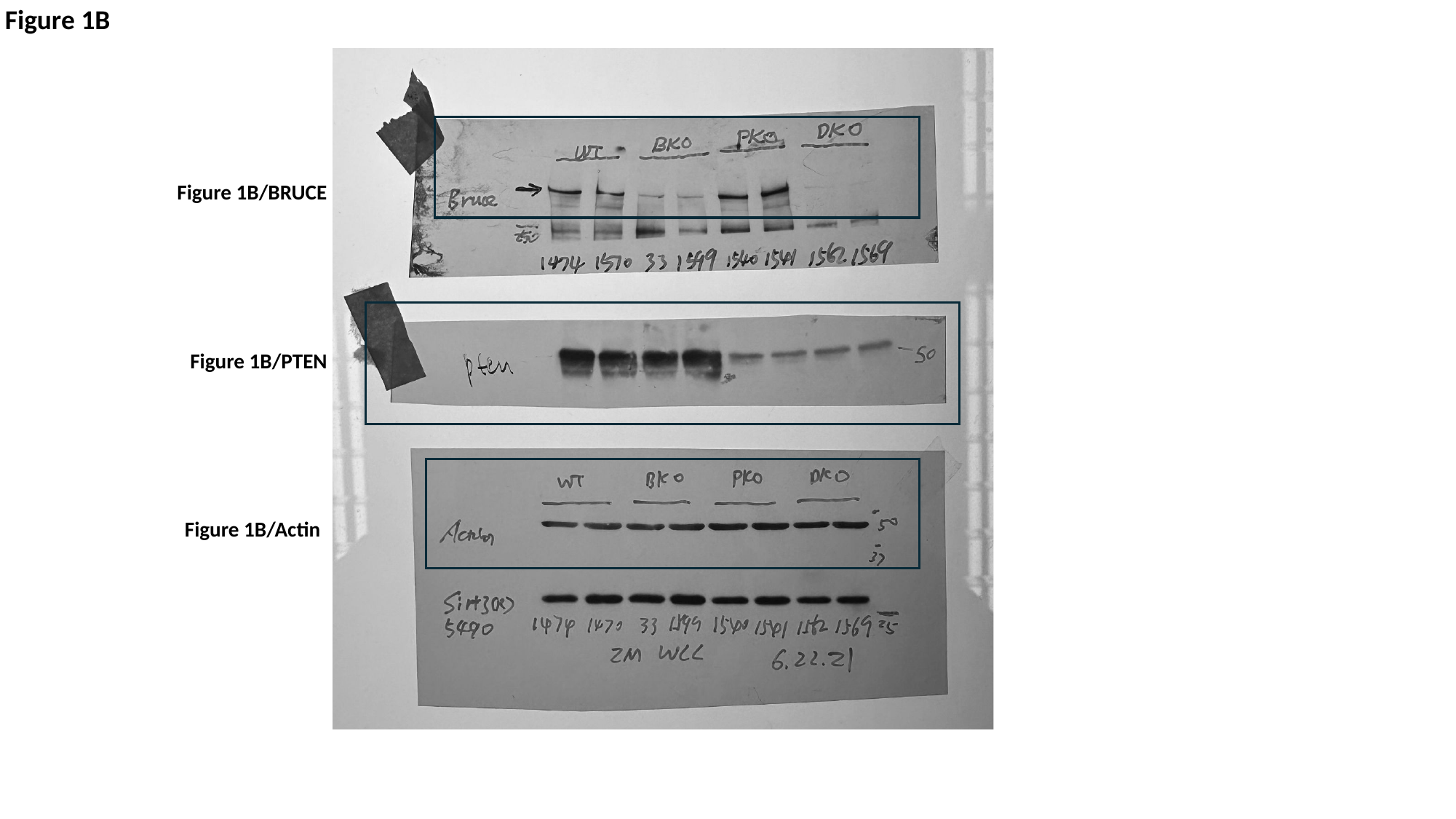

Figure 1B
Figure 1B/BRUCE
Figure 1B/PTEN
Figure 1B/Actin

## Slide 2
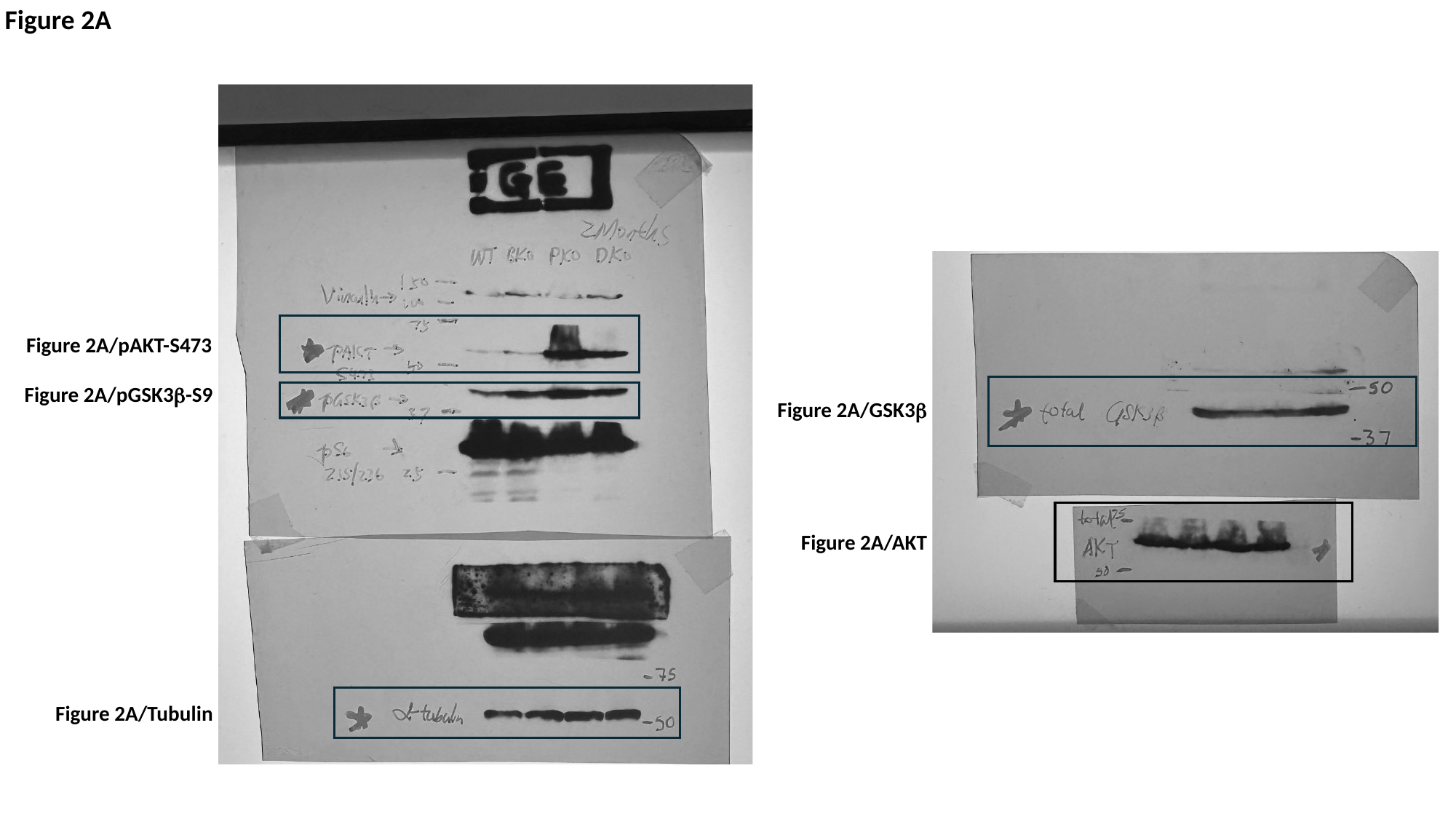

Figure 2A
Figure 2A/pAKT-S473
Figure 2A/pGSK3b-S9
Figure 2A/GSK3b
Figure 2A/AKT
Figure 2A/Tubulin

## Slide 3
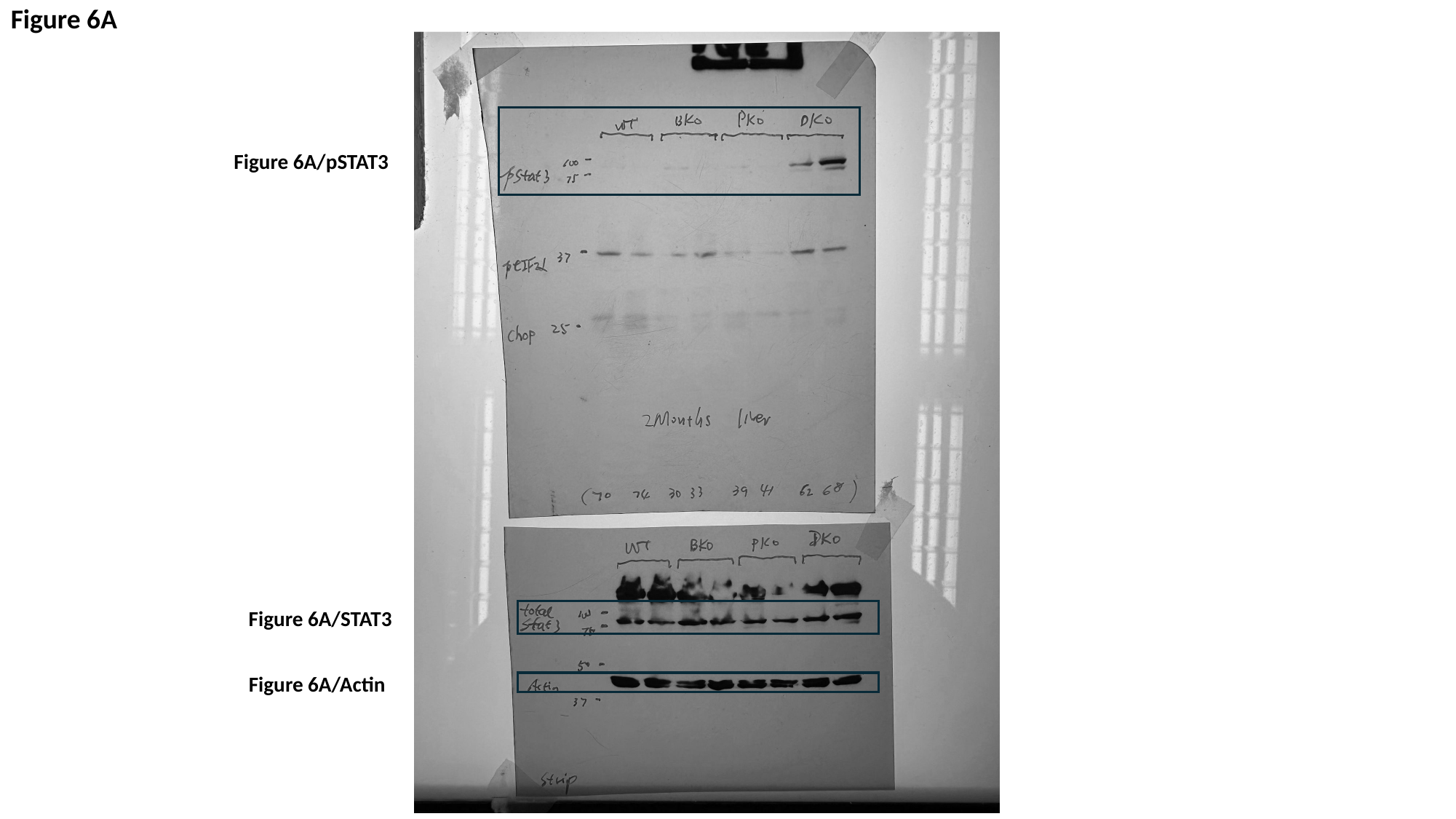

Figure 6A
Figure 6A/pSTAT3
Figure 6A/STAT3
Figure 6A/Actin

## Slide 4
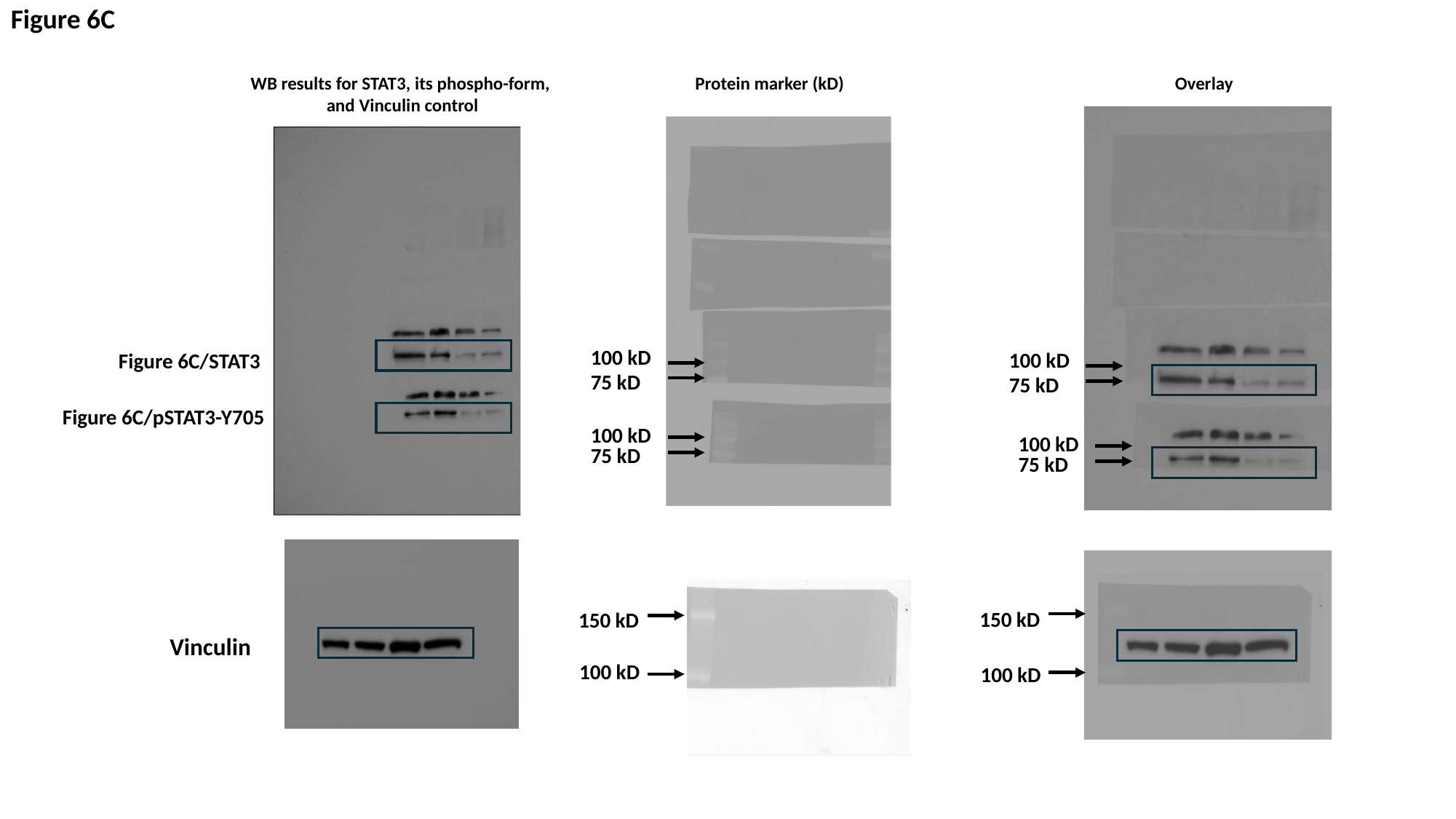

Figure 6C
WB results for STAT3, its phospho-form,
and Vinculin control
Protein marker (kD)
Overlay
100 kD
100 kD
Figure 6C/STAT3
75 kD
75 kD
Figure 6C/pSTAT3-Y705
100 kD
100 kD
75 kD
75 kD
150 kD
150 kD
Vinculin
100 kD
100 kD
